# Kinetics of Intermetallic Phase Precipitation in Manual Metal Arc Welded Duplex Stainless Steels

**DOI:** 10.3390/ma16247628

**Published:** 2023-12-13

**Authors:** Monika Orłowska, Krzysztof Pańcikiewicz, Aleksandra Świerczyńska, Michał Landowski

**Affiliations:** 1 Faculty of Metals Engineering and Industrial Computer Science, AGH University of Krakow, 30-059 Kraków, Poland; 2 Institute of Manufacturing and Materials Technology, Faculty of Mechanical Engineering and Ship Technology, Gdańsk University of Technology, 80-233 Gdańsk, Poland; aleksandra.swierczynska@pg.edu.pl (A.Ś.); michal.landowski@pg.edu.pl (M.L.)

**Keywords:** duplex steel, lean duplex steel, manual metal arc welding, intermetallic phase, chi phase, sigma phase, heat treatment

## Abstract

The article presents the influence of heat treatment on the kinetics of transformations in lean duplex LDX2101 steel and a weld made of standard duplex 2209 material, which was welded by manual metal arc welding. Changes in the microstructure, hardness, and magnetic phase content were analyzed after heat treatment was conducted at a temperature of 800 °C for a period ranging from 15 to 1440 min. Light and scanning microscopy, Vickers hardness measurements, and magnetic phase content measurements using a ferritoscope were used for the research. In the LDX2101 steel, the presence of δ-ferrite and γ austenite was identified and additional Cr_2_N nitrides were observed in the heat-affected zone. After heat treatment, the decomposition of δ ferrite into γ_2_ austenite and Cr_2_N nitrides was observed in both areas. In the case of weld made by the coated electrode in 2209 grade, a ferritic–austenitic microstructure with allotriomorphic austenite (γ_A_), Widmanstätten austenite (γ_W_), and idiomorphic austenite (γ_I_) and δ-ferrite area with “bee swarms” of fine precipitations of chromium nitrides Cr_2_N and non-metallic inclusions (NMIs) of slag, formed during the welding process, are observed in the as-welded state. After heat treatment, the presence of the χ phase (after 15 min of annealing) and the σ phase (after 120 min of annealing) was additionally identified. The kinetics of intermetallic phase evolution in welds made from 2209 material were presented. The obtained results of hardness measurements and metallographic tests were correlated, which allowed for a quick check of the precipitation processes on the used element.

## 1. Introduction

Difficult operating conditions of devices and working structures, e.g., in the energy, food, chemical, paper, and marine industries, require the use of materials that are resistant to environmental impacts and, at the same time, are characterized by high durability [1,2]. A common choice of designers in such situations are chromium-nickel corrosion-resistant steels with a two-phase structure (ferritic–austenitic), which demonstrate good corrosion resistance and higher mechanical properties than standard austenitic steels [3]. Typically, these steels are used in contact with aqueous solutions of chlorides and as a substitute for austenitic steels. However, among others, traditional austenitic steels have limited resistance to stress corrosion cracking in media containing chlorides. The material resistant to these conditions are corrosion-resistant ferritic steels but problems with weldability (grain growth in the HAZ) do not favor their use and nickel alloys have a high price, which also discourages investors. The above factors led to the creation of duplex ferritic–austenitic steels, which have good resistance to intergranular and stress corrosion cracking. The development of this group of steels, forced by the need to reduce costs and ensure an equilibrium structure and appropriate weldability, has led to the development of many types of duplex steels, which are currently divided into lean duplex (LD), duplex, super duplex, and hyper duplex. All species from these groups are characterized by a balanced structure of approximately 50% ferrite and the main classification criterion in this case is the chemical composition [3,4]. The welding of elements from this group of high-alloy steels with a complex structure requires meeting certain procedural conditions that reduce the risk to their weldability. The basic problems of weldability of duplex steels include the risk of loss of mechanical and corrosion properties resulting from the increase in the volume fraction of ferrite due to the impact of short welding thermal cycles and the tendency to form cold cracks (and other manifestations of hydrogen embrittlement) and hot cracks as well as precipitations of intermetallic phases, e.g., the sigma (σ) phase [2,3,5,6].

In work [3], Varbai showed the results of welding simulation tests using the Gleeble device, indicating a strong correlation between cooling conditions and the microstructural morphology and corrosion resistance of LD X2CrNiN22-2 steel specimens. Specifically, the author reported that the HAZ of the 2202 steel grade shows excellent weldability as, even at shorter cooling times (lower heat inputs), the austenite content remains above 30% on average. Yang and Ni also conducted advanced research on the structure and corrosion properties of LD stainless steel subjected to a simulated heat cycle, comparing them with the properties of classic 2205 duplex steel [7]. The objective of the article [8] was to present the influence of changes in the value of heat input on the mechanical properties of welded joints. The authors stated that low heat input resulted in higher ultimate strength and yield strength compared to the base material, while high heat input welded joints achieved slightly lower values. Elongation during the tensile test decreased by approximately 15% in both cases. The results of research on the structure–properties relationship of welded joints produced by the flux cored arc welding (FCAW) process indicate the formation of titanium- and silicon-rich oxides. However, their presence did not cause a significant change in the impact strength of the welded joints [9].

Duplex steels can be successfully joined using various arc processes (SMAW, MAG, FCAW, GTAW, and SAW) as well as laser, resistance, and friction processes [1,2,3,4,8,10,11]. Very good results are also reported in the case of making dissimilar joints of duplex steel with other iron alloys and even with non-ferrous metal alloys [10,12]. The SMAW process is still of great practical importance in the welding of corrosion-resistant steels as it is characterized by high technological and metallurgical versatility and satisfactory efficiency [8,13]. An additional advantage of the process is the possibility of using it in situ when repairs are necessary in difficult-to-reach locations. The most serious limitation to the wider application of this process is the human factor: training welders with the appropriate skills, awareness, and responsibility [13].

The welding parameters of ferritic–austenitic steels must be strictly controlled. The cooling rate should be low enough to allow complete transformation of austenite in the high-temperature HAZ area (so that the ferrite grains do not grow too large) and, at the same time, high enough to avoid the formation of precipitates in the low-temperature HAZ area [14,15]. Welding may disturb the proportions of the ferritic and austenitic phases in the joint, even to above 90% ferrite. In order to restore the balance between the contents of both phases, it is recommended to perform solution annealing. In such a case, post-welding heat treatment must be strictly controlled due to the risk of secondary austenite being released from the ferrite [16].

During the exposure to elevated temperatures, intermetallic phases χ and σ are precipitated from the solid solution. The chi (χ) phase is an intermetallic phase with a topologically close-packed (TCP) structure [17] found in the literature in a wide stoichiometric range from Fe_36_Cr_12_Mo_10_ [18] to Fe_35_Ni_3_Cr_13_Ti_7_ [19]. Due to the solubility of carbon in the χ phase, it was initially confused with M_18_C type carbide [20]. Its presence in the microstructure of ferritic–austenitic steels leads to increased hardness, brittleness, and reduced corrosion resistance [17]. The sigma (σ) phase is a non-magnetic intermetallic phase with a tetragonal crystallographic structure [17] with 32 atoms per unit cell [21], similar to the χ phase found in a wide stoichiometric range from Fe_59_Cr_41_ to Fe_49_Cr_51_ [22]. Its impact on the mechanical properties and corrosion resistance is similarly unfavorable to that of the χ phases [23].

The mentioned limitations in the weldability of duplex steel are still the main topic of many publications, which proves the relevance of the subject and the need to continue research aimed at eliminating threats to the operation of welded joints made of duplex steel. Therefore, in order to assess the kinetics of intermetallic phase formation in materials with a chemical composition corresponding to lean duplex (LDX2101) and standard duplex (2209 filler metal) steels, a welded joint was made using the manual metal arc welding process and heat treated at 800 °C for various times. Based on the research, a quantitative and qualitative assessment of precipitations was made, the kinetics of precipitation were described, and an indirect method for assessing the progress of transformation was proposed, which has not been reported. Correlating the results of mechanical and metallographic tests provides an innovative approach to the indirect assessment of changes related to the precipitation processes of intermetallic phases.

## 2. Filler Metal Selection

Table 1 lists filler metals intended for welding two-phase ferritic–austenitic steels according to ISO 14343-A [24].

The chemical composition of the filler metal in grade 22 9 3 N L (2209) is most similar to the standard duplex steel grade 2205 (UNS S31803) and is characterized by an average content of 22.5%Cr, 8.5%Ni, 3.25%Mo, and up to 2.5%Mn. In relation to grade 2209, the standard proposes three grades with a richer chemical composition and one with a poorer chemical composition. Grades 25 7 2 L, 25 9 3 Cu N L, and 25 9 4 N L have an average content of 25.5%Cr and an average content of 7%Ni and 2%Mo for 25 7 2 L and approximately 9.5%Ni and 3.25%Mo for the other two. These materials differ significantly in their copper content and the last one has the addition of tungsten. In the case of a material with a poorer chemical composition, namely 23 7 N L (2307), the chromium content is higher than in the 2209 alloy and amounts to an average of 23.5%Cr but the remaining components have a lower content, i.e., an average of 8%Ni and up to 0.8%Mo. The standard also allows binders with a different agreed chemical composition but their designation must be preceded by the letter Z. 

Leading manufacturers of welding consumables dedicate the grades of consumables available in the standard for welding particular groups of duplex steel. For example, the filler material 23 7 N L (2307) is indicated as suitable for welding lean duplex steels (UNS S32101, S32304, S32001), while the filler material 22 9 3 N L (2209) is indicated as suitable for welding standard grades of duplex (UNS S32205, S31803) but also suitable for welding lean duplex steel (UNS S32304, S32101).

Due to the poorer chemical composition of lean duplex steels, in order to utilize the economic potential, manufacturers rightly expect filler material producers to propose providing materials tailored to the basic materials. An example of such a proposal is a filler metal with a composition of 0.03%C–1%Mn–24%Cr–9%Ni–0.2%Mo–0.2%Cu–0.15%N with a PREn coefficient of 24 [25]. They have shown that from 2000 to 2009, the cost of new filler material compared to filler material 2209 dropped from 90% in 2000 to 70% in 2009, resulting in 30% savings.

## 3. Materials and Methods

The test material constitutes manual metal arc welded and multilayered butt joints made of lean duplex LDX 2101 grade steel (Werkstoffnummer 1.4162, X2CrMnNiN21-5-1, UNS S32101) with the dimensions 6.5 × 150 × 300 mm. The filler metal was covered electrode ELGA DUPLEX LP/E2209-17 with diameter ϕ3.2 mm in the grade EN ISO 14343-A: E 22 9 3 N L (1st filler material from Table 1). Welding was carried out manually on a Kemppi KMS 400 (Kemppi, Lahti, Finland) device and parameters were recorded using the ArcInfo system (Kemppi, Lahti, Finland). The parameters of the welding process are presented in Table 2. To calculate the heat input, the coefficient k = 0.8 according to EN 1011-1, was used. The welded joint was non-destructively tested and obtained the B quality level according to EN ISO 5817 [26].

Then, nine samples were cut from the welded joint, eight of which were annealed at 800 °C for 15 min, 30 min, 60 min, 120 min, 180 min, 240 min, 300 min, and 1440 min. Samples were analyzed in all characteristic areas of the welded joint.

The chemical compositions of the base material were according to the EN 10088-5 [27] standard, the filler metal was according to ISO 14343-A [24] standard, and optical emission spectroscopy (OES) analysis of steel and weld metal is presented in Table 3. 

The OES was performed with the Foundry Master-WAS Spectrometer (Hitachi, Tokyo, Japan). Observation of the microstructure was carried out with a Leica DM/LM (Leica, Wetzlar, Germany) light microscope (LM) using a bright field (BF) and a Phenom XL (Thermo Fisher Scientific, Waltham, MA, USA) scanning electron microscope (SEM) with backscattered electrons (BSE). The SEM-BSE images were excited by an electron beam with an accelerating voltage of 20 kV and a current of 10 nA at a working distance of 4 mm and a pressure of 1 Pa. Energy dispersive spectroscopy (EDS) analysis was used to analyze the chemical composition in the micro-area. The X-rays were excited by an electron beam with parameters similar to those used for SEM-BSE imaging. Metallographic tests were carried out in accordance with ISO 17639 [28] and the EDS analysis was conducted in accordance with ASTM E1508 [29]. For microscopic examination, the samples were mechanically grinded, polished, and etched. Mechanical grinding with water cooling was carried out on silicon carbide grinding papers with decreasing grit from 100 to 4000, with a constant load of 20 N and a disc rotation speed of 200 rpm. Mechanical polishing was carried out on a polishing cloth with the addition of an aqueous solution of ground aluminum oxide powder (deagglomerate AP-D Powder 0.1 μm, Struers, Copenhagen, Denmark) with a constant load of 5 N and a disc rotation speed of 50 rpm. The samples were electrically etched in a 20% water oxalic acid solution and 10% water NaOH solution (according to CR 12361 [30]). Hardness measurements were performed with a Zwick/Roell ZHU 187,5 universal hardness tester (Zwick Roell Group, Ulm, Germany) using the Vickers method with an intender load of 10kG (98.07 N) and dwell time 10 s, according to ISO 6507-1 [31] and ISO 9015-1 [32]. The content of the magnetic phase was verified using the MPD-100A ferritoscope according to ISO 8249 [33]. The charts and their analysis were made using Origin 2023b (OriginLab, Northampton, MA, USA).

## 4. Results and Discussion

### 4.1. Microstructure of the Base Material

**Figure 1:**
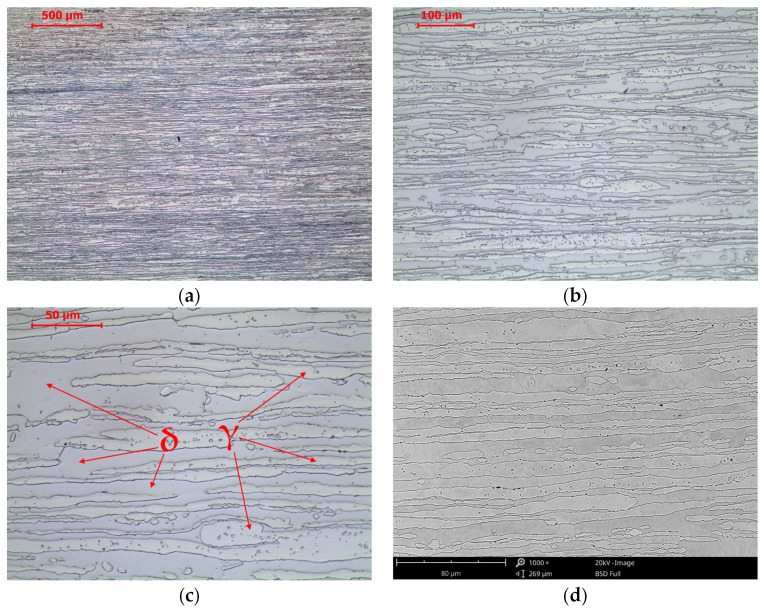
Microstructure of base material LDX 2101 steel in the as-delivery state. LM-BF (**a**–**c**) and SEM-BSE (**d**).

**Figure 2:**
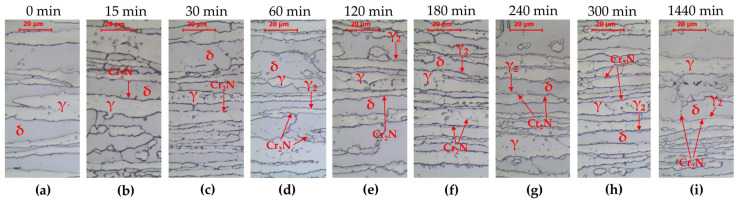
Microstructure of base material LDX 2101 as-delivery state (**a**) and after annealing at 800 °C: (**b**) 15 min, (**c**) 30 min, (**d**) 60 min, (**e**) 120 min, (**f**) 180 min, (**g**) 240 min, (**h**) 300 min, and (**i**) 1440 min. LM-BF.

The tested steel was characterized by a banded microstructure characteristic of products shaped by rolling (Figure 1a). A typical microstructure of the γ phase (austenite) distributed in the matrix of the δ phase (δ-ferrite) was observed. An analogous structure after hot rolling of lean duplex LDX2101 steel was observed by G. Ubertalli et al. [34], F. Iacoviello et al. [35], W. Gong et al. [36], F. Tehovnik [37] or S. Gudikandula, and A. Sharma [38]. Changes in the microstructure after annealing at 800 °C are shown in Figure 2. The phase equilibrium diagram for LDX2101 steel [37] indicates that at this temperature, chromium nitrides Cr_2_N and trace amounts of M_23_C_6_ carbide may also be present in equilibrium with austenite and ferrite. The appearance of precipitates is observed after 15 min of annealing (Figure 2b). This means that even a short time of holding at a temperature of 800 °C will predispose the release of chromium nitrides at the δ/γ interface. After annealing for 60 min, clear effects of the decomposition of δ-ferrite into austenite and the precipitation of nitrides and/or carbides at the δ/γ interface are already observed in accordance with the following reaction:

(1) $$\delta =\gamma_{2}+{\mathrm{Cr}}_{2}N+M_{23}C_{6}$$

**Figure 3:**
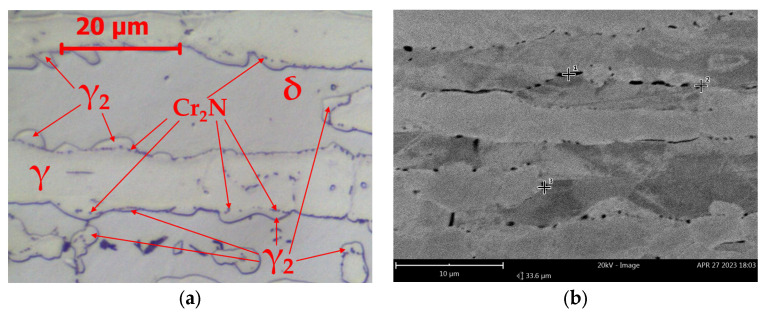
Microstructure of base material LDX 2101 after annealing 800 °C/1440 min: (**a**) LM-BF and (**b**) SEM-BSE.

With increasing annealing time, no significant microstructural changes were observed in LDX 2101 steel. After 1440 min (Figure 3), the microstructure consisted of δ-ferrite, primary austenite (γ), austenite (γ_2_) formed as a result of δ-ferrite decomposition and fine Cr_2_N precipitations. The characteristic feature of the layer of separated nitrides is their location on the primary δ/γ interface boundary. This is why they constitute a specific interface between γ austenite and γ_2_ austenite.

### 4.2. Microstructure of the Heat-Affected Zone

**Figure 4:**
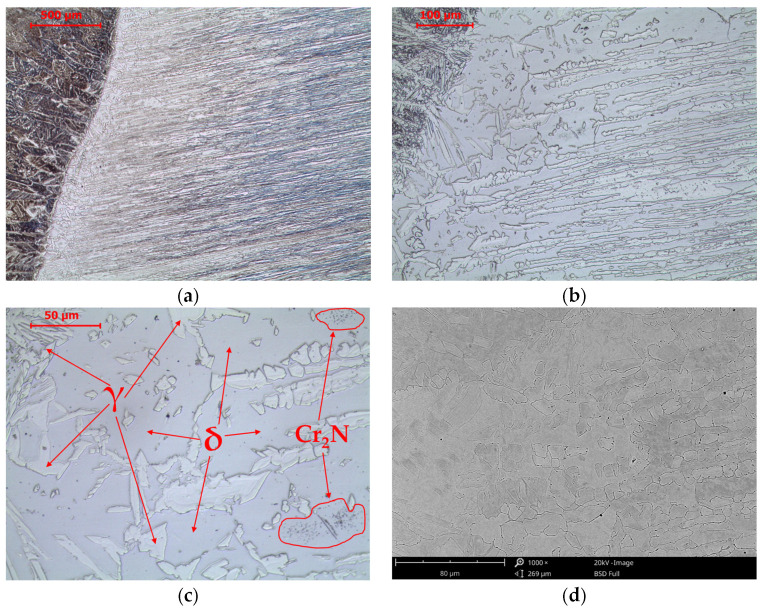
Microstructure of the heat-affected zone in LDX 2101 steel in the as-welded state. LM-BF (**a**–**c**) and SEM-BSE (**d**).

The microstructure of the heat-affected zone is divided into high-temperature and low-temperature heat-affected zones, which differ in grain size and the degree of defragmentation of the band-arranged austenite (Figure 4). The observed higher content of ferrite compared to austenite in the high-temperature heat-affected zone in the as-welded state results from the relatively high cooling rate, which is due to the input of a small amount of heat into the layer (Table 2). The welding process heats the material, creating a temperature gradient from the solidus temperature near the fusion line to the initial temperature in the area away from the fusion line. Heating the high-temperature heat-affected zone above the solvus line leads to the complete transformation of austenite into δ-ferrite. Once the heat source is removed, cooling begins. The faster the cooling rate, the more it inhibits the transformation of δ-ferrite into austenite and the greater the volume fraction of δ-ferrite after cooling to an ambient temperature. The so-called “bee swarms”, which are chromium nitrides, Cr_2_N, isolated as a result of rapid cooling from the existence of δ-ferrite. The solubility of nitrogen in δ-ferrite is high at high temperatures but decreases as the temperature decreases. As a result, they are released in the steel structure in the heat-affected zone after cooling.

**Figure 5:**
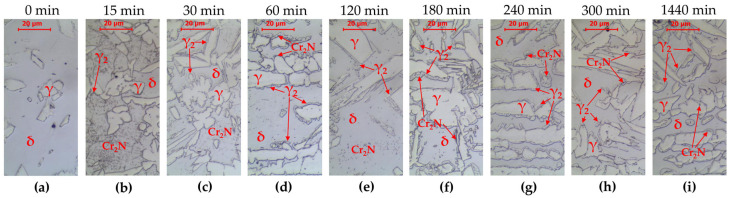
Microstructure of the heat-affected zone in LDX 2101 in the as-welded state (**a**) and after annealing at 800 °C: (**b**) 15 min, (**c**) 30 min, (**d**) 60 min, (**e**) 120 min, (**f**) 180 min, (**g**) 240 min, (**h**) 300 min, and (**i**) 1440 min. LM-BF.

Similarly to the base material, heat treatment at 800 °C does not significantly affect the phase composition of the heat-affected zone (Figure 5). Increased release of chromium nitrides Cr_2_N and δ-ferrite disintegration at the δ/γ interfaces are observed in accordance with Equation (1).

**Figure 6:**
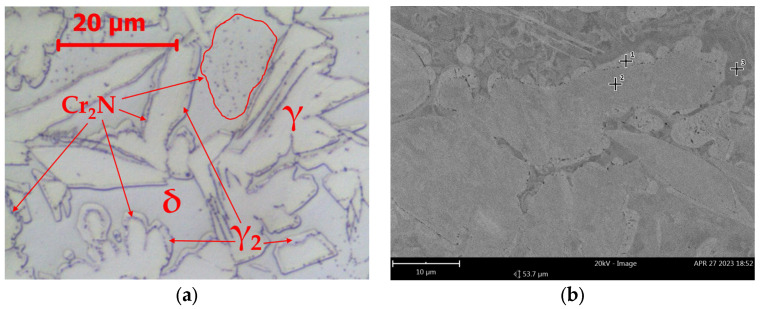
Microstructure of the heat-affected zone in LDX 2101 steel after annealing 800 °C: (**a**) 1440 min; LM-BF and (**b**) 120 min; SEM-BSE.

After 1440 h of annealing, a δ-ferritic–austenitic microstructure is observed (Figure 6, Table 4). Chromium nitrides, Cr_2_N, precipitate in the δ-ferrite and at the δ/γ interface boundary, which has become the γ/Cr_2_N/γ_2_/δ boundary due to the disintegration of δ-ferrite.

### 4.3. Microstructure of the Weld Metal

**Figure 7:**
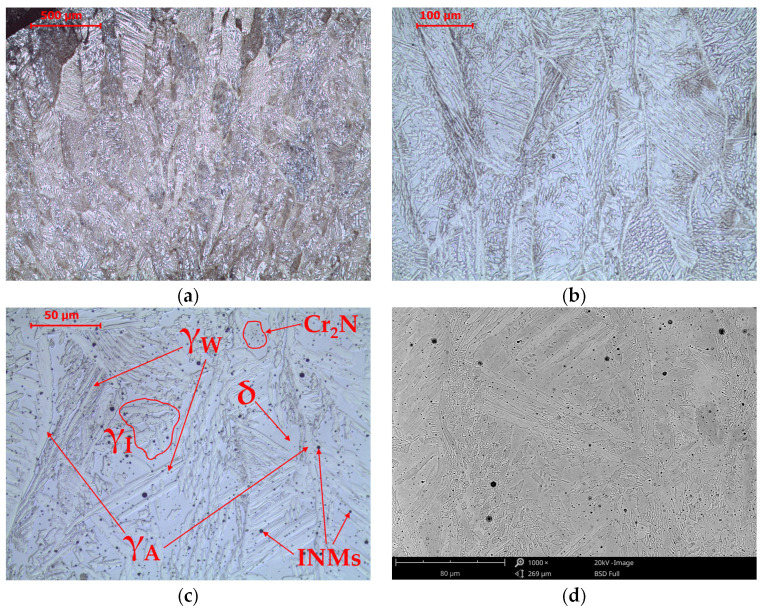
Microstructure of E2209 weld metal in the as-welded state. LM-BF (**a**–**c**) and SEM-BSE (**d**).

The microstructure in the weld area heated above the liquidus temperature is characterized by a typical casting structure (Figure 7). Ferritic crystallization of duplex steel determines the enrichment of ferrite-forming components in the crystallite cores and austenite-forming components on the crystallite boundaries. Passing through the solvus line during cooling begins the transformation of δ-ferrite into austenite. The place where the transformation begins is the crystallite boundary due to the lowest energy needed to nucleate a new phase and due to the enrichment of areas close to the crystallite boundary with nickel. In this area, at ambient temperature, allotriomorphic austenite (γ_A_) is observed. The emerging δ/γ interface becomes a place for further growth of allotriomorphic austenite (γ_A_) or, if there is a Kurdiumow–Sachs relationship between δ-ferrite and austenite, Widmanstätten austenite (γ_W_). The third morphological type of austenite is idiomorphic austenite (γ_I_). In the δ-ferrite area, “bee swarms” of fine precipitations of chromium nitrides Cr_2_N and non-metallic inclusions (NMIs) of slag formed during the welding process are also observed. The observed NMIs are oxy-sulfides, enriched in manganese, silicon, titanium, and calcium (Table 5). The share of other elements and, partly, of manganese and silicon is related to the characteristics of the testing method in which part of the signal comes from the matrix under the inclusion. The presence of inclusions is caused by the fact that the liquid slag did not have time to escape to the weld surface during solidification. Due to the fact that they are spherical and very small, these inclusions do not significantly impair the properties of the welded joint. The average volume fraction of fine slag inclusions (NMIs) in the weld is approximately 0.8%.

**Figure 8:**
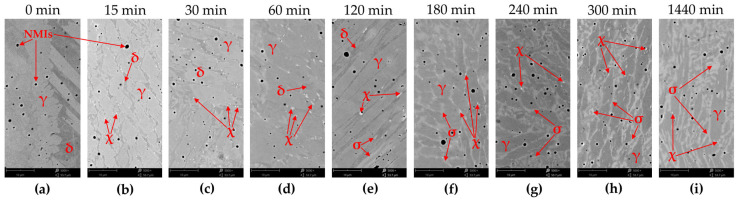
Microstructure of E2209 weld in the as-welded state (**a**) and after annealing at 800 °C: (**b**) 15 min, (**c**) 30 min, (**d**) 60 min, (**e**) 120 min, (**f**) 180 min, (**g**) 240 min, (**h**) 300 min, and (**i**) 1440 min. LM-BF.

**Figure 9:**
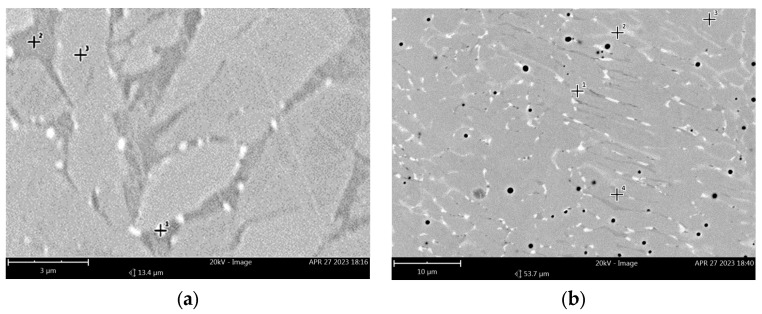
Microstructure of E2209 weld after annealing 800 °C: (**a**) 15 min. and (**b**) 1440 min. SEM-BSE.

The effect of heat treatment at a temperature of 800 °C on the microstructure is shown in Figure 8. After 15 min of annealing, bright single very small precipitates appear on the δ/γ interfaces. These precipitates are characterized by a high content of Cr (approximately 25% by weight) and Mo (approximately 14% by weight) and are probably the χ phase (Figure 9a, Table 6). The number of precipitates increases as the annealing time increases, up to 60 min. After 120 min of annealing, larger and slightly darker precipitates appear on the δ/γ interfaces compared to the χ phase precipitates. These precipitates contain a larger amount of Cr (approximately 31% by weight) and a smaller amount of Mo (approximately 7.5%) than the χ phase precipitates (Figure 9b, Table 6). This is probably the σ phase. With increasing time, there is an increase in the content of the σ phase and decreases in the content of the χ phase and δ-ferrite are observed. However, after 24 h of annealing, only austenite and the σ phase are observed in the microstructure. 

**Figure 10:**
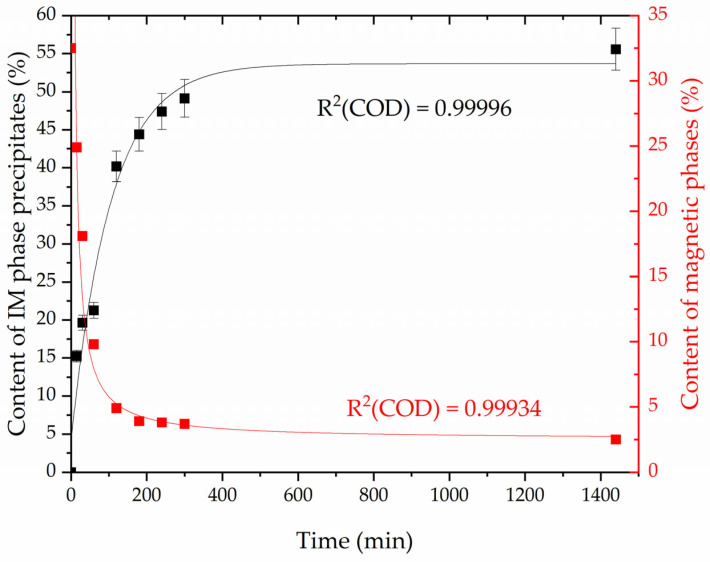
Dependence of the amount of intermetallic (IM) phase precipitates and content of magnetic phase in the weld metal on the annealing time at a temperature of 800 °C.

The trend of changes is confirmed by magnetic tests. As the content of intermetallic (IM) phases increases, the content of the magnetic phase, which is δ-ferrite, decreases. The change in the content of IM phases, assessed by metallography as a percentage of the surface, and the change in the amount of the magnetic phase measured with a ferritoscope are summarized in Figure 10.

An asymptotic curve with Equation (2) was fitted to the results of the dependence of the amount of intermetallic phase precipitates on the annealing time at a temperature of 800 °C using orthogonal distance regression. In this way, a fit of R^2^ (COD) of 0.99996 was obtained. The rational curve from Equation (3) was fitted to the results of the dependence of the amount of magnetic phase on the annealing time at 800 °C using orthogonal distance regression. In this way, a fit of R^2^ (COD) of 0.99934 was obtained. Detailed data are presented in Table 7.

(2) $$y=a\;-\;b\;\cdot \;c^{x}$$

(3) y=(1+cx)/(a+bx)

### 4.4. Hardness of the Welded Joint

**Figure 11:**
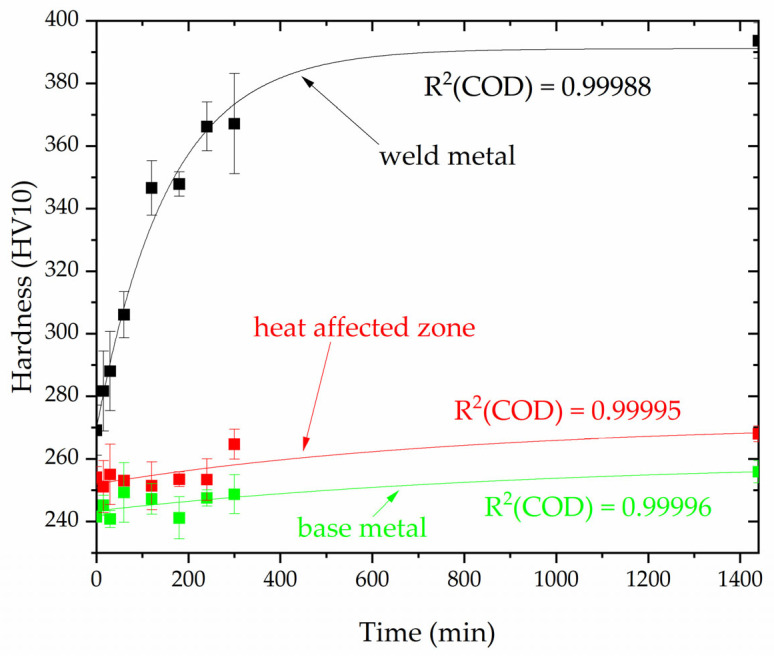
Dependence of the hardness on the annealing time at a temperature of 800 °C.

Hardness measurements (Figure 11) confirmed the observed trend of changes in microscopic and magnetic examinations. The hardness measurements showed a significant increase in weld metal hardness with an increasing annealing time at 800 °C. The asymptotic hardness increase demonstrates a steady rise after a short period of annealing. After 60 min, the hardness increased by an average of 37 HV10 and, after another 60 min, it increased by an average of 40 HV10. The subsequent increase in hardness was not as rapid, amounting to no more than 20 HV10 for the following 180 min and the next 1140 min. The asymptotic curve fitted using orthogonal distance regression is characterized by an R^2^ (COD) fit of 0.99988 and the parameters of Equation (2) for this case are listed in Table 8.

In the case of the base material (LDX2101 steel) and its heat-affected zone, the changes in hardness are not significant. A slight increase in hardness was noted as a function of the annealing time. In the heat-affected zone and the base material, the hardness increased by approximately 14 HV10 after 1440 min. The asymptotic curves fitted using orthogonal distance regression are characterized by an R^2^ (COD) fit of 0.99995 and an R^2^ (COD) fit of 0.99996, respectively, and the parameters of Equation (2) for these cases are listed in Table 8.

### 4.5. Discussion

The test results presented in Section 4.1, Section 4.2, Section 4.3 and Section 4.4 indicate differences in the occurrence of precipitation processes between lean duplex steel and a weld with a standard duplex chemical composition during annealing at a temperature of 800 °C.

In the case of LDX2101 steel, after 15 min of holding, precipitates of chromium nitride Cr_2_N are visible (Figure 2), located at the δ/γ interphase boundaries. However, there is conflicting information in this regard in the literature. Moon et al. [39] showed that in 25Cr–6Mn–3Ni–1Mo–3W–0.1C–0.34N lean duplex stainless steel at a temperature of 800 °C, secondary phases are separated at the δ/γ boundaries; however, no Cr_2_N nitrides were identified, only eutectic precipitates of M_23_C_6_ carbides. However, it should be borne in mind that the content of molybdenum and tungsten in this model alloy is higher than in the LDX2101 steel and both the increases in tungsten and molybdenum promote the formation and stability of the carbides of Cr-rich M_23_C_6_ [40]. Fang et al. [41] found in TEM investigation for LDX2101 steel that, after 30 min at a temperature of 800 °C, Cr_2_N precipitates are observed in the microstructure, while at a lower temperature (700 °C) Cr_23_C_6_ precipitates are observed. The low content of Cr and Mo and the addition of nitrogen were indicated as the reason for the absence of the σ phase [41]. Through tests using AFM, Ouali et al. [42] indicated the occurrence of nanometric M_23_C_6_ carbides after heat treatment at a temperature of 750 °C. On the other hand, Dandekar et al. [43] indicated that, after annealing at a temperature of 750 °C for less than 1440 min, the only secondary phase is Cr_2_N nitrides. It was also indicated that the molybdenum content at a level lower than 0.35% causes the nucleation and growth of the σ phase to be slow/delayed and the formation of M_7_C_3_ and M_23_C_6_ type carbides is the least possible. Finally, Tehovnik et al. [37] showed in TEM and SEM-EBSD observation that after heat treatment of 800 °C/120 min, chromium nitrides, Cr_2_N, and a small amount of M_23_C_6_ carbides are observed on the grain boundaries. It should be noted, however, that the tested alloy contained 0.32%Mo, which could facilitate the precipitation of this carbide. In the case of the alloy analyzed in this work, the molybdenum content was only 0.167% according to Table 3, which causes unfavorable conditions for the nucleation and growth of M_23_C_6_ carbides.

The second secondary phase formed as a result of reaction (1) in the LDX2101 steel is secondary austenite γ_2_, which becomes clearly visible in metallographic tests after being held at a temperature of 800 °C for between 30 and 60 min. The occurrence of γ_2_ austenite in LDX2101 steel is confirmed by numerous studies [37,41,42,43].

There is little information about microstructure changes in the heat-affected zone of LDX2101 steel and much less information about the influence of annealing. Analysis of microstructure changes in the heat-affected zone presented in the work of Ubertali et al. [34] confirmed the occurrence of precipitates of chromium nitrides Cr_2_N at the δ/γ interphase boundaries in the post-welded state. The occurrence of nitrides was also reported by Sun in [44]. Chromium nitrides, Cr_2_N, were observed in the HAZ even after welding underwater, i.e., under conditions of rapid heat removal [44]. This indicates the high susceptibility of LDX2101 steel, which is manifested by the required short residence time in a given temperature range for their precipitation in the ferrite-δ area.

The microstructure of the weld, made with a coated electrode producing a weld metal with the chemical composition of the standard duplex 2209 grade, consists of ferrite, austenite, and non-metallic inclusions (slag). Various morphologies of austenite are observed: allotriomorphic austenite (γ_A_), Widmanstätten austenite (γ_W_), and idiomorphic austenite (γ_I_). Their occurrence is consistent with literature reports for welds in grades 2209 [38,45,46,47,48,49,50,51,52], 2101 [53,54], 2205 [55,56,57], 2507, and 2304 [55]. Observations regarding the occurrence of non-metallic inclusions are also consistent with literature reports [58,59,60] and result from the specificity of the manual metal arc welding process. 

Unlike LDX2101 steel, welds with the chemical composition of standard duplex grade 2209 are characterized by high dynamics of the precipitation kinetics of new phases from the solution. After just 15 min of heat treatment at 800 °C, the precipitation of χ intermetallic phases is observed. These precipitates are located at the δ/γ interphase boundaries and, when observed using SEM-BSE, appear as bright areas. SEM-EDS analysis confirms the enrichment of these areas in molybdenum. This confirms the scattering of a larger number of backscattered electrons by heavier elements (molybdenum) relative to the others in the chemical composition (nickel, manganese, chromium, silicon, and carbon) and therefore their brighter appearance. On the other hand, it is indicated that the precipitation of the χ phase (bcc; Fe_36_Cr_12_Mo_10_) is promoted by the addition of molybdenum and the location of the precipitations and their shape correspond to literature reports [17,61,62]. As the annealing time increases to 120 min, an increase in the dimensions of the χ phase precipitates is observed. Extending the annealing time at 800 °C to 120 min also causes the release of another intermetallic phase, i.e., the σ phase. Due to its chemical composition and extensive morphological structure of the weld, the σ phase (Fe_100-x_Cr_x_, where x = 41 ÷ 51 [22]) is not as clearly visible in the SEM-BSE contrast. However, a detailed SEM-EDS analysis allows us to indicate with high probability that the areas with a different degree of gray represent the precipitation of the σ phase, resulting from the decomposition of ferrite in accordance with the reaction (4) [17]:

(4) $$\delta \to \gamma_{2}+\sigma$$

This reaction is also confirmed by a constant decrease in the content of the magnetic phase in the weld (Figure 10) due to the fact that austenite (γ_A_, γ_W_, γ_I_, and γ_2_), slag (NMI), and intermetallic phases (χ, σ) are non-magnetic. Annealing carried out on 2906 super duplex steel with molybdenum (2%) and nitrogen (0.38%) at a temperature of 800 °C showed that the share of intermetallic phases after 27 min of annealing increased from 0% to 9.2%, after 81 min of annealing it increased to 28.0%, and after 72 h to 37.5% [63]. The kinetics of phase separation, taking into account the differences in chemical composition and material condition (rolled material vs. weld) seems to correspond to that observed in grade 2209 (Figure 10). For welds of grade 2209, Kang et al. [45] showed that, after annealing at 800 °C and 850 °C for 30 min, the σ phase and small amounts of the χ phase and chromium nitrides Cr_2_N are present in the microstructure. The amount of magnetic phase at 850 °C, expressed by the ferrite number, was 1.9 FN (which approximately corresponds to the percentage of ferrite [64]). Therefore, a level was achieved that was very similar to that obtained in the research (Figure 10). Also, Antunes et al. [47] showed that after annealing of grade 2209 welds at a temperature of 700 °C for 50 and 100 h (3000 and 6000 min), γ austenite and χ and σ phases appear in the microstructure. No δ ferrite was found. However, it should be noted that the annealing time is much longer (over twice and over four times) than the maximum time in this experiment (1440 min). Additionally, the sequence of release of intermetallic phases at a temperature of 800 °C, suggested by Escriba et al. [65], was not confirmed, i.e., the release of the χ phase was preceded by the σ phase.

**Figure 12:**
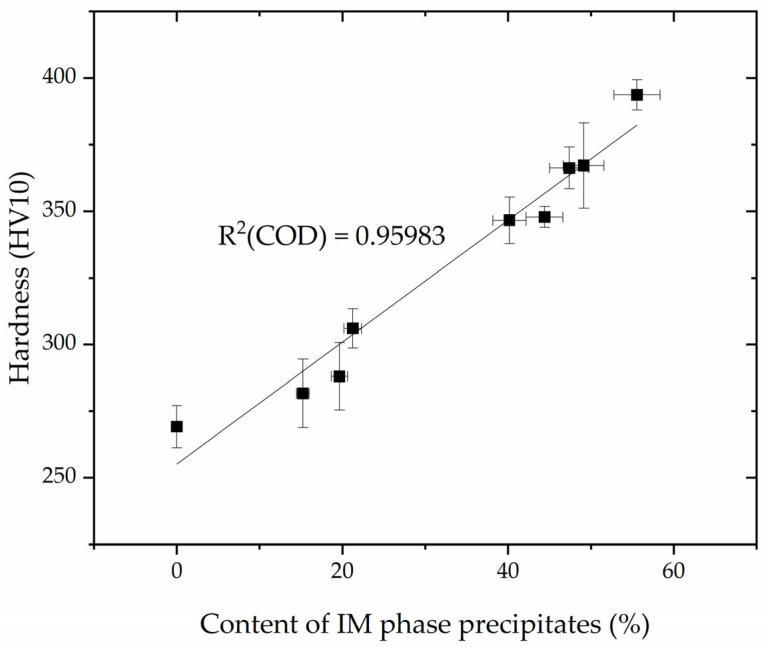
Dependence of the hardness on the annealing time at a temperature of 800 °C.

Based on the obtained results, the hardness measurements and the proportion of intermetallic phases in the weld were correlated. The asymptotic nature of the intermetallic phases and hardness curves with respect to annealing time curves leads to a linear relationship between these variables (Figure 12). 

The obtained regression line, described by Equation (5):

(5) $$y=a+b^{x}$$

where a = 255.11523 ± 6.57159 and b = 2.28943 ± 0.17703 has a relatively high correlation coefficient R^2^(COD) = 0.95983. It is possible to indirectly assess the proportion of intermetallic phases after heat treatment at 800 °C based on hardness measurements.

This information is valuable for laboratories without microscopic testing equipment but with hardness measurements tools. The established relationship enables the interpretation of structural changes and, consequently, material properties, aiding decisions on utilization or cessation.

## 5. Conclusions

The analysis of the microstructure, hardness, and amount of the magnetic phase in the manual metal arc-welded joint of LDX2101 steel with 2209 filler metal in an as-welded state and after heat treatment at 800 °C for 15 to 1440 min allowed the following conclusions to be drawn:

- LDX2101 steel and the heat-affected zone of LDX2101 steel at a temperature of 800 °C are not prone to the precipitate of intermetallic phases. During heat treatment, δ-ferrite decomposes into γ_2_ austenite and Cr_2_N nitrides;
- Welds made with 2209 filler metal are characterized by a ferritic–austenitic microstructure with Cr_2_N nitride precipitations and slag inclusions (resulting from the welding process used). Different austenite morphologies are observed, i.e., allotriomorphic austenite (γ_A_), Widmanstätten austenite (γ_W_), idiomorphic austenite (γ_I_), and austenite γ_2_ at the δ/γ interfaces. During heat treatment at a temperature of 800 °C, molybdenum-rich intermetallic χ phases are released (after just 15 min of annealing) and chromium-rich intermetallic σ phases (after 120 min of annealing);
- There is a strong correlation with R^2^(COD) = 0.95983 between hardness and the number of intermetallic phases in welds made with 2209 filler metal.

## Figures and Tables

**Table 1:** Chemical composition of filler metals according to ISO 14343-A [24], % mass.

Grade	C	Si	Mn	Cr	Ni	Mo	N	Other
22 9 3 N L	≤0.03	≤1.00	≤2.50	21.0–24.0	7.0–10.0	2.5–4.0	0.1–0.2	≤0.5 Cu
23 7 N L				22.5–25.5	6.5–9.5	≤0.8		
25 7 2 L				24.0–27.0	6.0–8.0	1.5–2.5	-	
25 9 3 Cu N L					8.0–11.0	2.5–4.0	0.1–0.2	1.5–2.5 Cu
25 9 4 N L					8.0–10.5	2.5–4.5	0.2–0.3	≤1.5 Cu, ≤1.5 W

**Table 2:** The parameters of the welding process.

Layer	Mean Welding Current I, A	Mean Arc Voltage U, V	Travel Speed v_w_, mm/min	Heat Input H, kJ/mm
1	62.7	19.1	145	0.40
2	106.9	19.4	190	0.52
3	109.7	17.2	170	0.53
4	109.0	20.1	175	0.60
5	89.7	17.8	240	0.32
6	84.6	18.5	235	0.32

**Table 3:** Chemical composition of base materials and weld, % mass.

Data Source	C	Si	Mn	P	S	Cr	Ni	Mo	Cu
OES (steel)	0.078 ± 0.001	0.657 ± 0.025	4.97 ± 0.021	0.0197 ± 0.0015	<0.005	21.2 ± 0.08	1.69 ± 0.04	0.167 ± 0.001	0.341 ± 0.005
EN 10088-5 [27]	≤0.040	≤1.000	4.0–6.0	≤0.004	≤0.015	21.0–22.0	-	0.1–0.8	0.1–0.8
OES (weld metal)	0.066 ± 0.003	0.850 ± 0.011	0.725 ± 0.033	0.0138 ± 0.004	0.0064 ± 0.0013	22.8 ± 0.09	9.61 ± 0.07	3.26 ± 0.014	0.141 ± 0.003
EN ISO 14343-A [24]	≤0.030	≤1.000	≤2.5	≤0.030	≤0.020	21.0–24.0	7.0–10.0	2.5–4.5	≤0.3

**Table 4:** Chemical composition of phases, SEM-EDS analysis.

Point of Analysis	Phase	Weight Concentration, %				
		Cr	Mo	Ni	Si	Mn
Figure 6b, point 1	γ_2_	17.14	1.25	2.08	1.10	6.70
Figure 6b, point 2	γ	21.11	0.64	1.32	0.87	6.10
Figure 6b, point 3	δ	22.19	0.50	1.11	0.98	5.60

**Table 5:** Chemical composition of example non-metallic inclusion (NMI), SEM-EDS analysis.

Concentration	O	Mn	Si	Cr	Fe	Ti	Al	S	Ca
Atomic	66.78	10.30	13.57	4.05	1.66	1.84	0.86	0.60	0.36
Weight	43.38	22.97	15.48	8.54	3.76	3.57	0.94	0.78	0.59

**Table 6:** Chemical composition of phases, SEM-EDS analysis.

Point of Analysis	Phase	Weight Concentration, %				
		Cr	Mo	Ni	Si	Mn
Figure 9a, point 1	χ	25.23	11.10	5.68	1.72	1.53
Figure 9a, point 2	δ	27.31	4.75	4.81	1.20	1.80
Figure 9a, point 3	γ	22.21	3.79	10.40	1.06	1.73
Figure 9b, point 1	χ	24.96	13.92	3.96	1.41	1.76
Figure 9b, point 2	σ	30.98	7.40	4.65	1.37	1.78
Figure 9b, point 3	γ	23.28	4.25	10.12	1.04	2.03
Figure 9b, point 4	δ	26.00	3.21	4.64	0.87	1.88

**Table 7:** The parameters of the equations.

Equation	a	b	c
(2)	53.651 ± 2.772	49.496 ± 0.991	0.991 ± 0.002
(3)	0	0.00305	0.00762

**Table 8:** The parameters of the equations.

Zone	Equation	a	b	c
Weld metal		391.065 ± 5.464	121.489 ± 6.579	0.994 ± 0.00008
Heat-affected zone	(2)	271.493 ± 10.686	19.545 ± 9.870	0.999 ± 0.001
Base material		259.455 ± 13.681	16.099 ± 12.771	0.999 ± 0.002

## Data Availability

Data are contained within the article.
